# Effects of mindfulness-based stress reduction for adults with sleep disturbance: a protocol for an update of a systematic review and meta-analysis

**DOI:** 10.1186/s13643-016-0228-2

**Published:** 2016-04-02

**Authors:** Seong Min Kim, Jeong Min Park, Hyun-Ju Seo

**Affiliations:** Department of Nursing, College of Medicine, Seonam University, Namwon, South Korea; Graduate School of Chosun University, Gwangju, South Korea; Department of Nursing, College of Medicine, Chosun University, 309 Pilmum-daero, Dong-gu, Gwangju, 61452 South Korea

**Keywords:** Mindfulness, Sleep disorders, Systematic review, Protocol

## Abstract

**Background:**

Sleep disturbance is a common and significant health problem that has been linked to decreased quality of life. Mindfulness-based stress reduction (MBSR) can be a potentially effective intervention for insomnia. In previous systematic review examining the effects of MBSR for people with sleep disturbance, the authors highlighted the need for additional well-designed randomized controlled trials (RCTs) to assess the effects of MBSR practice. Recently, several RCTs of the effectiveness of MBSR for individuals who have difficulties in sleep have been published. Therefore, the aim of this review is to update and synthesize evidence on the effects of MBSR for people with sleep disturbance or insomnia.

**Methods/design:**

We will search ovidMEDLINE, ovidEMBASE, Allied and Alternative Medicine, PsycINFO, the Cochrane Central Register of Controlled Trials, the Cumulative Index to Nursing and Allied Health Literature, and four representative Korean electronic databases including KoreaMed, the Korean Studies Information Service System, the Korean Medical Database, and the National Digital Science Library. Two reviewers will independently screen and select relevant studies. The Cochrane Risk of Bias tool will be used to assess risk of bias in the included studies. The primary outcomes will be defined as the change in sleep quality before and after the intervention as evaluated by the polysomnography or self-reported questionnaires such as the Pittsburgh Sleep Quality Index. If quantitative synthesis is not appropriate, a descriptive analysis might be undertaken.

**Discussion:**

Many published primary studies have investigated the positive effects of MBSR on sleep quality; however, there are no systematic reviews and meta-analyses synthesizing the evidence of up-to-date research on the effects of MBSR for sleep problems. The review findings will aid the general population and healthcare providers in making informed decisions on evidence-based intervention selection for sleep disturbance or insomnia.

**Systematic review registration:**

PROSPERO CRD42015027963.

**Electronic supplementary material:**

The online version of this article (doi:10.1186/s13643-016-0228-2) contains supplementary material, which is available to authorized users.

## Background

Sleep disturbance is recognized as a public health concern. Approximately 12.2~39.4 % of general population drawn from different countries were affected by symptoms of insomnia, such as difficulty initiating or maintaining sleep, waking up too early, and in some cases, having non-restorative or poor quality of sleep [1–4]. An estimated 50 % of older adults claimed to have difficulty initiating and maintaining sleep [5]. Sleep problems can lead to long-term physical and mental disorder, and linked to psychiatric conditions such as depression [6], anxiety [7], mood disorders [8], and dementia [9]. So, sleep disturbance deteriorate an individual’s quality of life and puts a significant economic burden on society [10]. Reduced productivity resulting from chronic insomnia in the US workforce has been estimated to cost $63.2 billion annually [11]. An additional $32 billion is spent by US consumers each year in the “sleep market” (e.g., hypnotics, sleep masks, and white noise devices) [12]. Therefore, treatment and management for sleep disturbances is a critical part of caring for people with sleep difficulties and insomnia symptoms.

Mindfulness-based stress reduction (MBSR) has been used as a complementary and alternative medicine therapy for alleviating stress and anxiety, and has been shown to play a role in alleviating symptoms of insomnia. MBSR is based on the Buddhist philosophy and was created by Jon Kabat-Zinn [13]. Kabat-Zinn defines mindfulness as “paying attention in a particular way: on purpose, in the present moment, and nonjudgmentally” [13]. This refers to “know awareness mind as” without the intervention of the subjective thoughts and feelings. Improving the mental and physical health through them and promotes insight into the meaning of life and physical changes leading to tranquility and peace of mind [14]. One approach to addressing insomnia that is rooted in addressing cognitive arousal is mindfulness-based training [15]. This practice allows individuals to focus on the present and let go of the thoughts, beliefs, and emotions that create stress [16]. In addition, it allows individuals to focus on the mental and physical states that lead to a positive response to sleep, and to avoid reacting in a negative way to sleep disturbances [15]. Thus, insomnia might be improved through an MBSR training program [16].

In previous systematic review evaluating the effectiveness of MBSR for people with sleep disturbance, there was some evidence to suggest that MBSR might improve sleep and decrease sleep-interfering cognitive processes [17]. However, due to the lack of standardized outcome measures of studies included in the review, a meta-analysis was not undertaken. And the authors of the review concluded that the well-designed randomized controlled trials (RCTs) are needed to elucidate the effects of MBSR practice. Recently, several RCTs of the effectiveness of MBSR for individuals who have difficulties in sleep have been published [18–21]. Therefore, the aim of this systematic review is to update and evaluate evidence on effectiveness of MBSR for peoples with sleep disturbance or insomnia.

## Methods/design

The systematic review and meta-analysis will be conducted in accordance with the Preferred Reporting Items for Systematic Reviews and Meta-Analyses (PRISMA) guidelines [22]. The following protocol has been reported in accordance with the PRISMA-P guidelines (Additional file 1). The protocol has been registered with the PROSPERO International Prospective Register of Systematic Reviews (CRD42015027963).

### Selection criteria

#### Study design

RCTs will be included without date and language restriction.

#### Population

All adults (at least 18 years old) regardless of health conditions were included, excepting shift-workers and travelers. It was not required to be diagnosed with sleep disorders.

#### Intervention

The MBSR programs will comprise standardized programs lasting for several weeks, including 6- to 10-week courses of 1- to 2.5-h sessions, or 1-day intensive training sessions. We will also include daily home practice programs [23]. Programs will need to be conducted by certified mindfulness teachers and delivered to an individual or group either face-to-face or online. The mindfulness practices covered will include mindful sitting meditation, mindful eating, appreciation meditation, friendly or loving-kindness meditation, mindful walking, and mindful movement [24]. We will include all studies utilizing MBSR, allowing for slight deviations from the original practices developed by Kabat-Zinn [13]. However, other mindfulness mediation practices such as mindfulness-based cognitive therapy, Vipassana, acceptance and commitment therapy, or Zen will be excluded from the review. In other words, only MBSR programs were of interest.

#### Comparators

The control group can be either passive (i.e., waitlist) and/or active (e.g., receiving other standard care or sleep hygiene education) [18–22].

#### Outcomes

We will assess the following outcome measures based on analyses of the data obtained in the included trials. Studies lacking standardized outcome measures (e.g., where primary outcomes are demonstrated using only graphs) will be excluded from the quantitative data analysis. The primary outcomes will be defined as the change in sleep quality before and after interventions. Objective sleep quality is measured by polysomnography or wrist actigraphy [19, 21]. Patient-reported outcome measures were total sleep time, sleep efficiency, sleep onset latency, and wake after sleep onset from sleep diaries, or self-reported questionnaires such as the Pittsburgh Sleep Quality Index and Insomnia Severity Index [18, 20]. The secondary outcomes considered will be sleep-related daytime impairments (depressive symptoms, anxiety symptoms, perceived stress, and fatigue symptoms) and quality of life [18, 20, 21].

Trials in which MBSR was used in a combination modality (e.g., in combination with different meditation techniques or hypnotics) will be excluded if the treatment effect of MBSR could not be isolated.

### Search strategy

The following databases will be searched for relevant studies from their inception of database until October 2015: ovidMEDLINE, ovidEMBASE, Allied and Alternative Medicine (AMED), PsycINFO, the Cochrane Central Register of Controlled Trials (CENTRAL), and the Cumulative Index to Nursing and Allied Health Literature (CINAHL). Manual searches for additional studies will also be performed by reviewing the reference lists of relevant studies. We will also search for studies in four representative Korean databases, including KoreaMed, the Korean Studies Information Service System (KISS), the Korean Medical Database (KMbase), and the National Digital Science Library (NDSL).

Additional file 2 presents the full list of search terms that will be used, and the search terms were adapted for the other databases. Other relevant trials will be searched for by manually screening the reference lists of trials and relevant review papers identified in the initial searches. In addition, the corresponding authors of selected studies will be contacted to supplement incomplete information.

Ongoing clinical trials will be searched for on Clinical trials.gov (https://clinicaltrials.gov/ct2/home) and the World Health Organization International Clinical Trials Registry Platform (WHO ICTRP; http://apps.who.int/trialsearch/).

### Study selection

**Fig. 1:**
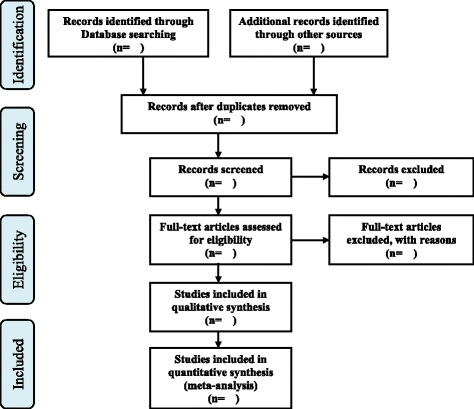
A PRISMA flow diagram

The reference software program EndNote^®^ (EndNote X7, Thomson Reuters, New York, USA) will be used to manage articles and remove duplicate references. Two independent reviewers (KSM and PJM) will select the studies according to the eligibility criteria. Selection process will be as follows: the first, titles and abstracts of the retrieved records will be screened to identify relevant studies. In cases of inconsistencies, the full text will be read. The second, full text of the articles selected in the first stage will be assessed and scrutinized. Disagreements will be discussed and resolved by consensus, if necessary, a third reviewer (SHJ) will be consulted. The details of the selection process will be presented in the Preferred Reporting Items for Systematic review and Meta-Analysis (PRISMA) flow diagram (Fig. 1) [25]. The excluded studies will be listed in a supplementary file along with our reasons for exclusion.

### Assessment of risk of bias in included studies

The reviewers will independently assess the methodological quality of the included studies using the Cochrane Risk of Bias (RoB) tool [26]. The RoB tool comprises seven specific domains addressing five types of bias: random sequence generation (selection bias), allocation concealment (selection bias), blinding of participants and personnel (performance bias), blinding of outcome assessment (detection bias), incomplete outcome data (attrition bias), selective outcome reporting (reporting bias), and other sources of bias. We will judge the risk of bias of each study as “high,” “unclear,” or “low” according to the results of the RoB tool. Any disagreement will be resolved via consensus or consultation with a third reviewer.

### Data extraction

Before data extraction, we will devise a standardized form containing the specified outcomes via a discussion among all reviewers. Two reviewers will then extract data independently using this standardized form. Any disagreements regarding extracted data will be resolved via consensus with a third person. The data extraction form will include the study design, country of publication, number and characteristics of participants, details of the MBSR program, follow-up periods, and primary/secondary outcomes. Where reported data are insufficient or ambiguous, reviewers will contact the corresponding authors by e-mail requesting additional clarification or information.

### Data synthesis

Dichotomous data will be reported as the risk ratio (RR) with 95 % confidence intervals (CIs). For continuous data, we will use the mean difference (MD) to measure the treatment effects with 95 % CIs. However, if the outcome variables were measured using different scales, we will use the standardized mean difference (SMD) with 95 % CIs.

A meta-analysis will be carried out where appropriate to synthesize data from similar studies using Review Manager (V.5.3) and STATA 10.0 (Stata Corp., College Station, Texas). Using the DerSimonian method and the Laird random-effects model, the pooled estimates of the weighted mean difference (WMD) or SMD with 95 % CIs will be calculated for continuous outcomes and RRs with 95 % CIs for dichotomous outcomes [27]. We will assess heterogeneity of treatment effects across studies using the *I*^2^ and the Q-statistic [28]. An *I*^2^ value of >50 % will be considered an indication of substantial heterogeneity [29], and if heterogeneity is observed, we will conduct a subgroup analysis. Where possible, we will perform a subgroup analysis (1) by type of sleep disturbance (e.g., the severity of sleep disturbance or insomnia) and (2) by duration of MBSR (e.g., short-term treatment of 8 weeks or long-term treatment of more than 8 weeks). A sensitivity analysis will be considered for studies that can be grouped based on their methodological quality and funding sources. A Funnel plot will be used to detect any publication bias if more than 10 studies are included in this analysis [28]. Additionally, we will perform Egger’s statistical test to detect any possible publication bias (using the criterion of *p* < 0.1) [30]. If a quantitative synthesis is not appropriate, a narrative synthesis will be conducted to interpret the data.

### Quality of evidence and dissemination of findings

We will use the Grading of Recommendations, Assessment, Development and Evaluation (GRADE) approach to judge the quality of evidence for outcomes of interest. This approach considers the following aspects to assess the quality of a body of evidence: study design, the risk of bias, imprecision of results, inconsistency across studies, application of results to the population of interest, and likelihood of publication bias [31]. Quality will be rated as high, moderate, low, or very low. The results will be disseminated through peer-reviewed publication and international conferences.

## Discussion

MBSR is widely applied in both clinical populations and healthy individuals to alleviate stress and stress-related health conditions such as cardiac diseases [32] and long-term physical conditions [33], as well as to promote health [34, 35]. Sleep disturbances are considered a significant medical and public health concern. However, it remains unclear whether MBSR can be used to alleviate sleep difficulties [17]. As there are several RCTs on the effects of MBSR on sleep problems [36–38], we thought it is necessary to provide an up-to-date systematic review of the evidence on MBSR. Therefore, the results from the review proposed herein will be carefully interpreted according to study quality, the content of the MBSR program, and the clinical importance of the outcome measures used in the included studies. The findings of this review will aid both the general population and healthcare providers to make informed decisions on evidence-based intervention alternatives for sleep disturbance or insomnia.

### Limitations

Because our database searches will be limited to published articles, bias might be introduced through the exclusion of unpublished data from thesis and conference proceedings abstracts. This publication bias might inflate the treatment effect estimates because studies with desirable or significant results are more likely to be granted publication. If we suspect any publication bias, Duval’s trim and fill method will be used to correct for it [39].

## Additional files

Additional file 1: PRISMA-P checklist. (DOCX 19 kb) Additional file 2: Sample search strategy for ovidMEDLINE. (DOC 28 kb)

## Abbreviations

MBSR: mindfulness-based stress reduction
RCT: randomized controlled trial.
