# Supplementary material for: Effects of mindfulness-based stress reduction for adults with sleep disturbance: a protocol for an update of a systematic review and meta-analysis
Source: Syst Rev. 2016 Apr 2;5:51. doi: 10.1186/s13643-016-0228-2 (PMC4818935; doi:10.1186/s13643-016-0228-2)
Supplement: Additional file 2: — Sample search strategy for ovidMEDLINE. (DOC 28 kb) [file 13643_2016_228_MOESM2_ESM.doc]

Additional file 2: Sample search strategy for ovidMEDLINE

| **ovidMEDLINE search strategy** |
| --- |
| 1 exp mindfulness/  2 exp meditation/  3 MBSR.mp.  4 MBCT.mp.  5 MBTI.mp.  6 mindfulness-based stress reduction.mp.  7 mindfulness-based cognitive therapy.mp.  8 mindfulness-based therapy for insomnia.mp.  9 cognitive.ab.ti.  10 (mind-body adj3rela*).mp.  11 or/1-10  12 exp sleep disorders/  13 sleep disturbance.mp.  14 exp sleep/  15 sleep*.mp.  16 insomnia.mp.  17 or/12-16  18 11 and 17 |
| **The terms were adapted for the other databases:**  ovidEMBASE  CINAHL  PsychINFO  AMED  CENTRAL  KoreaMed  KMbase  KISS  NDSL |
